# Pyorrhea Alveolaris

**Published:** 1896-01

**Authors:** B. F. Arrington

**Affiliations:** Goldsboro, N. C.


THE
AMERICAN JOURNAL
—OF—
DENTAL SCIENCE.

Vol. xxix. Baltimore, January, 1896. No. 9.

ARTICLE I.

### PYORRHEA ALVEOLARIS.

BY DR. B. F. ARRINGTON, GOLDSBORO, N. C.

*Mr. President and Gentlemen of the Maryland State Dental Association:*

In looking over a recent issue of the *Dental Cosmos* and reading a lengthy paper pertaining to the treatment of pyorrhea alveolaris, written by a prominent member of the profession, giving his treatment in full, very elaborate, displaying unquestionable ability as a writer, and extensive familiarity with remedies; but so seriously deficient in correct features of a right conception of the disease and conservative treatment for relief and cure, as I view the subject, I have decided to discuss this much abused subject, and will, instead of mentioning more than seventy-five remedies, as in the paper above referred to, for treatment of the disease, name less than half a dozen (quite sufficient) including *forceps* and *scalers*, important factors in treating cases of long standing.

In looking over some dental journals one rainy afternoon not long since, I recorded in less than two hours' time more than a hundred remedies suggested and advocated by sundry writers and practitioners of dentistry in the treatment of pyorrhea. I paused to reflect and was amazed at so extravagant a mention of remedies, and so dissimilar in properties, the like never known or heard of before in the practice of dentistry or medicine. I felt not only amazed, but must candidly confess I was disgusted and wished truly that better judgment should prevail to guide in practice. But when I read the paper in *Dental Cosmos*, and noted the mention of more than seventy-five remedies by one practitioner (and he a professor in a dental college) I considered the climax as to enumeration of remedies had been reached and it was time to call a halt. You can imagine my surprise and non-acceptance of such a line of remedies and treatment for a single disease, a disease simple and controllable, possibly of more frequent occurrence than any disease known and treated by the dental or medical profession. My greater surprise was that editors (practicing dentists) in charge of dental journals will permit articles so extreme and impractical to be published. Certainly no good can be effected by them. Less writing and discussing for notoriety, and more for practical benefit would doubtless profit us more. The paper in question enumerating so many remedies is ably and well written, and valuable in some respects. but is far short of the mark in a practical point of view in reference to treatment of pyorrhea. The various theories (as to the cause of the disease) set forth and advocated by sundry parties are almost as numerous as the remedies proposed in treatment, varying from slivers of tooth picks implanted between teeth, to gouty diathesis, diabetes, systemic debility, displacement of uterus, etc. A well known professor (a ready writer) in one of the dental colleges in Philadelphia sets forth as causes of the disease as follows: "Systemic predisposition, combined with decided induration of tooth tissues or other local irritant (sanguinary calculus, caries, or necrosis of process edge and gum pouches)." A conglomerate mass of absurdities, and far from correct as to cause. The causes enumerated, except systemic predisposition, are almost universally consequent upon the disease, and when not, certainly not the cause. Pyorrhea, like most other well defined diseases, has one cause and one only, not many, as some seem to think. It is such unreasonable guesswork and wild theorizing as this, heralded by prominent men in the profession, that leads astray and causes so much controversy. Unfortunately for those to be instructed in dentistry, all professors in dental colleges are not as knowing and practical as might be, and ought to be. By such teachers much is done that has to be undone. Some contend that pyorrhea is a constitutional disease, and requires for cure, constitutional treatment; others contend that it is only a local disease, and requires only local treatment, and there are others, a small minority, I am glad to say, who contend that it is constitutional and that there is no cure short of removal of the teeth; weak theory and censurable practice. Some of the best men in the profession argue and insist that the disease commences at apex of roots of teeth, while others, equally able and experienced in the practice of dentistry, contend that it commences at the gingival border or margin around the necks of the teeth. Why should there be such difference of opinion on the subject? Were I to attempt to quote authors, as to cause and treatment, it would make this paper too lengthy and of no additional value. If the literature published on the subject in dental journals during the past twelve or fifteen years could be compiled and published in book form it would make several volumes of thousands of pages (mostly impractical and valueless matter) speculative theory as to cause, and impractical ideas as to treatment. The profession would, in all probability, be disgusted with the compilation as a whole. After all that has been said and written concerning the disease, the profession stands as far apart and non-agreed as to cause and treatment to-day as it did ten years ago. There has been too much said about a little matter, and discussions and controversy continues, and no one knows or can predict when or where it will end. The great amount of theorizing as to cause of the disease and the extravagant list of remedies (embracing hundreds) recommended in treatment, exceed anything to be found in dental or medical literature, and instead of establishing facts to base action upon, has created confusion and doubt, and is misleading junior members of the profession, some even doubt the propriety of attempting to treat the disease. I know men in the profession who have never treated a single case, and invariably advises against treatment, saying to patients that the disease is incurable, and if treated and checked, it will soon return again. So much for false theory and false teaching of older heads, (some *professors* in *dental colleges.*)

When a practitioner of dentistry proclaims that Pyorrhea is incurable and advises non-interference, it is evidence of ignorance and non-progress, inexcusable, considering the progressive period in which we live. I know of a professor in a dental college, (presuming to impart instruction for the preservation of teeth, who proclaims that extraction of teeth is the only sure remedy for cure of of pyorrhea, quite as extreme and short of the true line of conservatism that should guide in teaching and practice, as the author of the paper here mentioned, who enumerates remedies so extravagantly, or the third professor who names so many causes of the disease. Such extremes and hitting short of the mark, evidence weakness and something wrong, there is evident fault somewhere, and it should be investigated and remedied, if possible. Such men cannot be regarded as safe guides and teachers of dental practice. Few educators in dentistry, a medium ground, always conservative, is wisest and safest, best for those instructed and for patrons they are to severe. What would be thought of a practitioner of medicine or a surgeon who would advocate and advise the amputation of an arm or leg infested with chronic ulcers, because remedies applied and reapplied for weeks or months failed to cure, or should mention for use in treatment remedies more than "three score and ten ?" I very much question if any medical journal in the country would give publicity, with favorable endorsement, to any such unreasonable theory and practice. It is sadly to be regretted that so much has been written and discussed pertaining to the disease, cause and treatment, unless something more definite as to both, had been established and agreed upon.

Pyorrhea alveolaris, as we term it for want of a more appropriate name, (gingivitis preferable), is a diseased condition of the gums, originating in, and always in its incipiency, confined to the gingival border, never at the edge of roots of teeth, or anywhere on roots, from apex to margin of alveolar process. The disease is local, well defined and easy to diagnose. I reiterate without fear of successful contradiction, that it always, without exception, commences in the gingival border, therefore is misnamed when called pyorrhea alveolaris. The alveolar process is never involved until the disease, commencing at the border or margin of gum has considerably advanced. Pus does not originate in the alveolar process, (no one can show it and prove it), nor is there any waste of the process only as the effect of pus generated in the soft tissues. In the early stage of the disease there are neither pus nor granular deposits. The first deposits are granular and always confined to the necks of the teeth; then follows pus formation and discharge, which more frequently than otherwise continues until the teeth loosen and drop out, or the disease is treated or cured. I have never known teeth to loosen until after cessation of granular formations around the necks of the teeth and the formation of solid cake deposits on the roots. Always the consequence of a disturbed, abnormal state of soft tissues.

A patient, female, aged 68, from whose mouth was extracted months ago the five teeth here exhibited, had been troubled with the disease more than thirty years. When I first saw her, twenty-seven years ago, and detected an unhealthy state of the gums, there were granular deposits, more or less, around the necks of all of her teeth. The deposits were never removed by means of instruments, but, as you see, passed away, possibly through use of the brush. She assured me when I extracted the teeth that no dentist had ever advised her to have the deposits removed since I advised her to do so, in 1867; said she had used mouth washes freely, recommended by dentists and physicians to cure the scurvy as they termed it, and that possibly she had expended several hundred dollars for such remedies, and without any good result. My observation has confirmed the impression on my mind that the granular deposits formed around the necks of teeth, will, sooner or latter, waste away and the surface become smooth, as you see demonstrated on the specimens, but the solid cake deposit, which is a second deposit later on in the disease, (very much of the character of deposits found on apex of roots) and never till the granular formation ceases, but when formed, is there to remain, never wastes or obliterates as the granular but increases, to the destruction of alveolar process and loss of teeth.

"Sanguinary deposit at or near the apex of roots is more difficult to treat than the disease in question. In the case of sanguinary deposit (not the effect or result of pyorrhea) the pus is formed deep down in the socket and progresses for exit, to alveolar margin. Whereas in pyorrhea the pus formation commences at the margin of alveolus and by degrees invades the entire process, leading to loss of the same and loss of the teeth. Every case of gum waste or recession of gum tissue we meet with is not pyorrhea; far from it. In a genuine case of pyorrhea that has advanced beyond the incipient stage, we find tumefaction of gum, discharge of blood or pus (often both) on application of pressure. In cases of recession of gum (not caused by pyorrhea) we find the reverse, almost universally. Instead of inflammation, tumefaction and discharge of blood and pus from the gum, as in pyorrhea, the gum is often pale, contracted and shriveled. I have seen many such cases, with loss of alveolar process and recession of the gum quite to the apex of the pal tal roots of the superior molars, with freedom from tenderness under pressure, (and no blood or pus visible.) In such cases the teeth never loosen as they do in pyorrhea, nor is there fetid breath. In well developed cases of pyorrhea, far advanced, deposits are always to be found and blood and pus freely flow. In gum waste or recession of gum, there is no deposit, or very little, ever to be found, nor is there discharge of blood or pus. Very unlike derangements of gum tissues. Pyorrhea, if neglected, becomes offensive and loathsome. Gum waste progresses slowly, without painful, disagreeable or offensive features, and never to the extent of loss of teeth " In case of sanguinary formation located at or near the apex of roots, extraction is the only sure remedy, and should not be delayed. It is impossible to effect cessation of pus discharge in pyorrhea so long as a minute particle of cake deposit remains on any portion of a root in contact with soft tissues. When the deposit has advanced to the extent seen on specimens nothing short of extraction will give relief. I contend, and have for many years, that when from the effect of the disease there has been destruction of two-thirds or three-fourths of the alveolus, teeth should be extracted, for there is no line of treatment by which the alveolar process can be restored and teeth made firm in sockets, nor can the gum ever be made to adhere again to the teeth, but if there is sufficient alveolar support, and all deposit is removed, the gum, after proper treatment, will assume healthy action and contract closely and firmly around the teeth, and if persistently manipulated with a tooth brush of suitable size and shape, and occasional finger pressure, will continue normal and perform a healthy function through life, unless weakened and rendered abnormal by some other disease than pyorrhea.

Some dentists do not recognize or acknowledge the difference between genuine pyorrhea deposits and ordinary calcareous deposits. The specimens submitted demonstrate the difference. Pyorrhea deposits never attach to enamel and crowns of teeth, but always to the cementum, while the ordinary salivary calculus adheres to enamel, and sometimes completely coats and covers crowns to the thickness of a sixteen or eighth of an inch, and is easy of removal compared with pyorrhea deposits.

Pyorrhea, like any other disease, is amenable to treatment; and can be cured, and when cured will remain so, if instructions as to care, cleanliness, free use of brush, etc., are carefully observed. There is no justification for so much talk about the disease not being curable, such ideas are extremely absurd, should and must be discarded, and all must treat for cure, and the results will prove convincing and satisfactory. What one man in the profession can do, others can, on the same line of procedure, if they will. I have not, nor do I presume to say that the line of treatment I pursue alone will cure the disease, but I do say emphatically, it will cure nineteen times in twenty. I have experimented considerably with various remedies recommended, but have never obtained as good results as with the remedies and treatment I will name and recommend in the interest of humanity.

As there is such diversity of opinion as to possibility of cure of this much discussed and abused disease, it may to some seem presumptious and egotistical in me to profess to be able to cure the disease with few remedies and but little trouble, and as effectively in a large percentage of cases, as any disease known to man, but there is no presumption or egotism in the matter. I make this statement on the subject as to treatment and cure to assist, in a plain, practical way, my professional brethren, if possible, and encourage them to regard the disease as curable, and to proceed on a conservative and effective line of treatment, trusting that favorable results may be the reward of their efforts, made in the right direction.

The first step in treatment is to test the strength of the teeth in their sockets, and if any are to be extracted (all should be that cannot be made comparatively firm in socket), extract before commencing to remove deposits. Thoroughness in the removal of deposits is all important in treatment, and to secure perfect success, *smooth edge scalers* must be used, such as I send for careful inspection, five of a set of ten I use, points varying in shape and size. Of the five, with those marked one, two and three, I remove at least nineteen-twentieths of all deposits. You perceive the edges are smooth and can do no injury to surface of root, alveolar process or soft tissues surrounding. To lacerate the gingival border (entire) and produce new action if possible, is important for cure. The removal of deposits prevents continuation of the disease, but does not prevent return of it. Soft tissues below and away from gingival border should be preserved as free from injury as possible; also the alveolar process, which is never in fault and has no part in the disease but to suffer loss, and is often seriously affected as the effect of a cause always originating in the gum border, which increases by degrees and creates the cause of alveolar trouble. Remove the cause and the effect will abate and the alveolus will speedily take on healthy action, always will, if deposits are thoroughly removed and *sulphuric acid* is generally applied. The practice advocated by some, to chisel, scrape and burr away the alveolar process (any portion of it) is absurd. Sulphuric acid properly applied is better for weakened and softened alveolus than chisels, burrs and scrapers.

I have never chiseled and scraped the process in treatment of the disease more than two or three times, and then it was not imperative for cure. When you think the last vestige of deposit has been removed, probe carefully again and again to make sure of completeness. In some extreme cases it requires from two to three hours' persistent effort for removal of deposits; when this has been accomplished, then with brush of proper size (small) and shaped to suit, brush heroically with diluted C. P. sulphuric acid and pulverized pumice (not finest) varying according to age and extent of disease, from one of acid to ten, fifteen or twenty of water. Make it a point, universally, to fret and lascerate the margin of gum with brush and pumice, with a view to new and healthy action. Do not fear injury to surface of teeth from the effects of the acid. No injury ever does or can result. As evidence of the slight effect upon enamel or cementum, I send for inspection a tooth which was kept in mixture of one of acid to ten of water, twenty-four hours.

Much good and no harm can come of the free use of sulphuric acid in treatment of the disease. It is seldom necessary to apply it more than one time for care. After the use of brush, acid and pumice, press the gums well with the finger, then apply to the gums freely camphophenique (full strength) and instruct the patient to use the brush, same as was used in the treatment, forcibly and systematically, several times daily for a week or two, specially after meals and before retiring at night, and to follow the brushing with finger pressure until the gums are healthy and firm, Advise the patient to return to the office the next day after treatment or the day following, and for several days, for careful inspection, probing and removal of any particles of deposit that may be detected, and to see if instructions as to brush and finger pressure are respected.

Some patients will neglect to carry out instructions, if not watched and encouraged to persevere. It is important in treatment and really essential, to use the brush forcibly and fret the gingival border to bleeding, if possible, for several days after removal of deposits. Applications of campho phenique (full strength) to the gums two or three times daily for several days will prove beneficial. In the incipient stage of the disease, before granular deposits are formed around the necks of the teeth, the free use of acid and pumice with brush is all that is requisite to check and obliterate the disease. It is at that stage as truly pyorrhea as in more advanced stages, when there are deep pus pockets, cake deposits on roots and teeth loosened to loss. It commences as pyorrhea, progresses and ends as pyorrhea, is pyorrhea all the way through, from commencement to cure or final loss of teeth. One line of treatment is requisite for cure, viz. : that above stated. If faithfully tried and persevered in, good results will always be realized. In cases as we meet with them, cure can be effected in from five to ten or fifteen days.

Such are my convictions, based upon experience and careful observation of results during many years' practice. I have made a specialty of the treatment of this disease for some years and only state as to treatment and results what I know to be facts, facts that will be realized and appreciated as such by any unprejudiced. fair-minded practitioner of dentistry, who will faithfully experiment for results on the line of treatment above indicated. The idea that *various causes* as above enumerated, can and do produce the disease in erroneous, and as ridiculously absurd as for physicians to say (some do) that this or that disease runs into typhoid fever, or that typhoid fever is the consequence of a broken or otherwise injured limb, or any other physical injury. Typhoid fever is a pointedly marked and distinct disease of well defined features and characteristics, and never the result of other diseases or physical injury. So it is with pyorrhea. No man can produce it by any injury he may inflict upon gum tissue or parts adjacent. It is spontaneous and truly an independent disease, having its well defined marks and running its course if let alone. In its incipiency it is recognizable and much more strongly marked and outlined than many other diseases, and if let alone will slowly advance to destruction of alveolus and loss of teeth. There is but one class of persons [the toothless) free from its ravages. It is of as frequent occurrence in early life, after the age of twelve or fourteen as in later years, but the cake, or sanguinary deposit (as termed by some) is never so well developed, and teeth but rarely loosen from effect of the disease before the age of thirty or thirty-five.

As to cause we are yet ignorant, and may grope in darkness on the subject a great while before the shadows are dispelled and the true light of cause shines forth. Physiologists and pathologists must investigate and settle the question. I am inclined to the opinion that the *saliva* contains the cause of the trouble. It is more reasonable than any other theory I can venture. After treatment and cure, the daily use of a tooth-brush corresponding to sample exhibited is requisite and occasional finger pressure will aid healthy preservation of the gums and serve as a safeguard against return of the disease. The size, shape and quality of a brush for daily use is an important matter. To cleanse the teeth well, and do no detriment to soft tissues, is to be considered in the selection and use of a brush. Large brushes with stiff bristles, closely set, are objectionable and are always more hurtful than beneficial.

I am impressed with the idea that a very large percentage of all the cases of pyorrhea I have ever seen, that could be successfully treated, the single line of treatment above stated, simple, plain and practical would suffice. The fewer the remedies, the better, so a good result is obtained. The advocacy of and sanction of a hundred remedies or more, for treatment of a single disease is contrary to all reason and radical in the extreme, and can but tend to bewilder the minds of young practitioners and cause many to question and doubt their ability to treat the disease at all. So much speculative theorizing as to cause of the disease and treatment, has doubtless done more harm than good, for there is no evidence of advance and a general line of treatment, truly defined, established.

I am recently very definitely convinced on one fact in relation to pyorrhea. It is not contagious or transmissible from one person to another. I have watched carefully for results for more than a quarter of a century, and I am now thoroughly convinced that there is no such thing as transmitting from parent to child, or from any person to another under circumstances. I have known several families residing in the country, embracing each parents and five or six children, where one or both parents were subject to the disease, well defined, and all would use one co-partnership toothbrush (a disgusting practice) until worn out, then another and so on, as needed, and not a child after a lapse of ten or fifteen years ever evinced the slightest feature of the disease. In one case in point, the husband, very loving and affectionate, fond of kissing his wife and children, was subject to the disease for more than thirty years (a typical case), losing by waste of process tooth after tooth, until but few remained in his mouth, and never did wife nor any of the children show signs of the disease. He (the husband) enjoyed good health, and he assured me more than once, he never suffered a moment from indigestion and experienced no particular discomfort from the disease.

I will mention one more case which I watched carefully for results for eighteen years, anticipating evil results from a censurable practice, but was disappointed. Mrs. —— died leaving a boy child about six or eight months old, who was taken charge of and cared for by its grandmother, and an old aunt, both subjects of pyorrhea well advanced, many teeth very loose, gums inflamed and swollen and blood and pus discharging freely under slightest pressure. The child was the pet of the family, and was tenderly cared for, first one then the other, grandmother and aunt feeding it, and for months the process of feeding was as follows: One or the other would chew and pulp the food in their mouths, then put it in the mouth of the child, so the food was prepared and administered until the child was able to masticate for itself. I advised against such a course and informed them of the chances and very great probability (as I then thought, do not now) of transmitting the disease to the child, but they heeded not. The child survived and grew up to manhood years and stature, a splendid specimen of physique and blessed with as healthy gums and sound teeth as could be desired. Should the young man be troubled with pyorrhea (quite possible) after this, would any contend that the disease was transmitted through food pulped and administered by grandmother and aunt? I could state other cases almost as extreme and quite as convincing to unbiased minds that the disease is not transferable by any process of contact or through any channel of conveyance of disease.

The disease has many peculiarities, and will oridinarily if not treated, run its course, and in a large majority of cases, will hold on during life or until the teeth involved loosen and drop out. All persons troubled with the disease do not suffer alike. Some do not experience any discomfort or inconvenience from the disease, while others suffer greatly and fail in health. Dentists should not be over-exercised concerning the cause of the disease. Physiologists and pathologist must and will settle the question, and possibly very soon, if they will look to the *saliva* during incipient stages of the disease and work on that line for facts that will be convincing. The important consideration for practitioners of dentistry is to learn how to diagnose correctly and treat for cure. If we will concern ourselves less about cause and direct our efforts in preparation to treat successfully pyorrhea or any other type of disease that may present for treatment, it would be wiser and more in line with our province of action, and doubtless best for our patients. All this theorizing and discussing as to cause, and advocacy of innumerable remedies for treatment is of no special benefit to dentists or patients, but is often mystifying and hurtful. To investigate cause of disease is one science, and to treat for cure is another, quite apart, separate and distinct, and if we attempt to couple them, we confuse and fail of best results. Cases known or not, let us treat to cure, and cure we can.—*Dental Cosmos* and *Southern Dental Journal.*

---

ARTICLE II.

### DR. PAINE'S LOCAL ANESTHETIC.

